# Advances in molecular characterization of myeloid proliferations associated with Down syndrome

**DOI:** 10.3389/fgene.2022.891214

**Published:** 2022-08-10

**Authors:** Jixia Li, Maggie L. Kalev-Zylinska

**Affiliations:** ^1^ Blood and Cancer Biology Laboratory, Department of Molecular Medicine and Pathology, University of Auckland, Auckland, New Zealand; ^2^ Department of Laboratory Medicine, School of Medicine, Foshan University, Foshan, China; ^3^ Haematology Laboratory, Department of Pathology and Laboratory Medicine, Auckland City Hospital, Auckland, New Zealand

**Keywords:** leukemia, acute myeloid leukemia, acute megakaryoblastic leukemia, transient abnormal myelopoiesis, Down syndrome, epigenetics, genomics, GATA1 mutations

## Abstract

Myeloid leukemia associated with Down syndrome (ML-DS) has a unique molecular landscape that differs from other subtypes of acute myeloid leukemia. ML-DS is often preceded by a myeloproliferative neoplastic condition called transient abnormal myelopoiesis (TAM) that disrupts megakaryocytic and erythroid differentiation. Over the last two decades, many genetic and epigenetic changes in TAM and ML-DS have been elucidated. These include overexpression of molecules and micro-RNAs located on chromosome 21, *GATA1* mutations, and a range of other somatic mutations and chromosomal alterations. In this review, we summarize molecular changes reported in TAM and ML-DS and provide a comprehensive discussion of these findings. Recent advances in the development of CRISPR/Cas9-modified induced pluripotent stem cell-based disease models are also highlighted. However, despite significant progress in this area, we still do not fully understand the pathogenesis of ML-DS, and there are no targeted therapies. Initial diagnosis of ML-DS has a favorable prognosis, but refractory and relapsed disease can be difficult to treat; therapeutic options are limited in Down syndrome children by their stronger sensitivity to the toxic effects of chemotherapy. Because of the rarity of TAM and ML-DS, large-scale multi-center studies would be helpful to advance molecular characterization of these diseases at different stages of development and progression.

## Introduction

Myeloid leukemia associated with Down syndrome (ML-DS) is a unique category of acute myeloid leukemia (AML) most often of the megakaryoblastic subtype (i.e., acute megakaryoblastic leukemia (AMKL), formerly known as AML-M7 (Singh et al., 2017). The term ML-DS also includes an antecedent myelodysplastic syndrome (MDS)-like phase. There is no biologic or prognostic difference between MDS (blasts 5–19%) and AML (blasts ≥20%) in Down syndrome (DS) (Lange et al., 1998), therefore this distinction is not being made for ML-DS in the current pathologic classification (Arber et al., 2016).

ML-DS is frequently preceded by transient abnormal myelopoiesis (TAM), a unique myeloproliferative disorder affecting megakaryocytic and erythroid lineages. TAM is a pre-leukemic condition characterized by reduced platelet and increased leukocyte counts, and the presence of blasts in the peripheral blood. TAM diagnosis requires the presence of *GATA1* mutations together with increased blasts and/or certain clinical features (in particular hepatosplenomegaly) in a neonate with constitutional trisomy 21, which can be mosaic (Arber et al., 2016). TAM may be indistinguishable from ML-DS but there is a wide spectrum of clinical presentation, ranging from asymptomatic to a stormy course and fatal outcome. Typically, TAM presents in neonates 3–7 days after birth but it may present within 2 months from birth (Singh et al., 2017). Overt TAM (blasts >10%) occurs in approximately 10–15% of DS neonates, but a further 10–15% may have *GATA1* mutations detectable only by sensitive methods with no clinical or hematologic manifestations (i.e., silent TAM) (Roberts et al., 2013). Most patients with TAM recover spontaneously within 3 months, but some require cytotoxic therapy. Unfortunately, despite initial TAM resolution, 20–30% of children progress to ML-DS within 4 years (Bombery and Vergilio, 2014).

TAM is extremely rare in neonates without DS but such cases have been well documented (Apollonsky et al., 2008; Tsai et al., 2011; Ono et al., 2015; Yuzawa et al., 2020; Panferova et al., 2021). The molecular pathogenesis and clinical outcomes of TAM in neonates without DS are similar to those with DS (i.e., DS-like). These patients acquire trisomy 21 and *GATA1* mutations in the TAM clone (Yuzawa et al., 2020; Panferova et al., 2021). In addition, *GATA1* mutations may be germline, as recently reported in familial childhood cases of TAM/AMKL, highlighting a unique functional cooperation between these lesions that may be independent of the order of their acquisition (Hasle et al., 2022). The rates of early death and leukemic progression of TAM in non-DS and DS children are similar, emphasizing the importance of making the diagnosis of DS-like TAM to assist appropriate patient management (Yuzawa et al., 2020). Rare cases of TAM without *GATA1* mutations feature in the literature. However, this may be due to technical limitations, in particular prior to the use of sensitive next-generation-sequencing methods (Panferova et al., 2021), small disease clones, or the lack of appropriate diagnostic samples if the condition is not suspected at presentation (Aksu et al., 2020). The expanding use of sensitive sequencing technologies will make the diagnosis of DS-like TAM easier in the future, which should advance our knowledge about this extremely rare condition.

ML-DS often presents with a period of thrombocytopenia reflecting a prodromal MDS-like phase (Lange et al., 1998). ML-DS is characterized by the expansion of megakaryoblasts, frequent bone marrow fibrosis, and the presence of *GATA1* mutations in the blasts that drive expression of a truncated (short) GATA1 protein (GATA1s) (Hasle et al., 2008). The median age of patients with ML-DS is 1–1.8 years (Gamis et al., 2003; Bhatnagar et al., 2016). Majority of patients with ML-DS (72%) also carry other cytogenetic changes in addition to trisomy 21 (Forestier et al., 2008; De Souza et al., 2017). The contribution of these changes to disease development and progression is the subject of active research.

**FIGURE 1:**
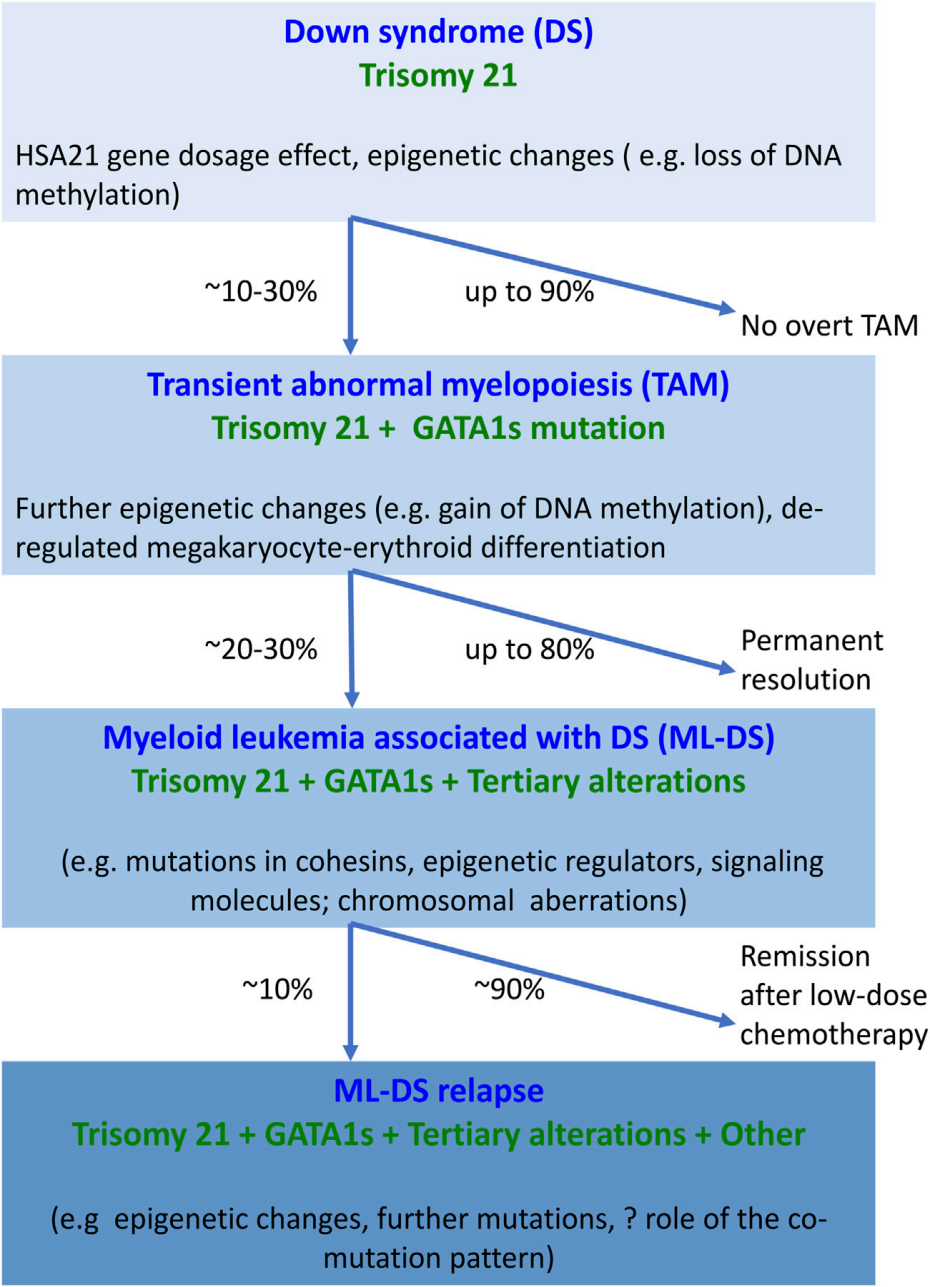
Overview of molecular changes reported at different stages of myeloid proliferation associated with Down syndrome. Trisomy 21 alone disturbs hematopoiesis through the increased dosage of HSA21-located genes and alterations in the epigenome, resulting in increased megakaryopoiesis. The combination of trisomy 21 and GATA1s causes expansion of megakaryocytic progenitors. Progression of TAM to ML-DS requires the interaction of GATA1s with additional somatic mutations and chromosomal structural abnormalities. Little is known about the molecular landscape of refractory or relapsed ML-DS. Abbreviations: DS, Down syndrome; GATA1s, GATA1 short; HSA21, human chromosome 21; ML-DS, myeloid leukemia associated with Down syndrome; TAM, transient abnormal myelopoiesis.

ML-DS pathogenesis is understood to follow a multistep clonal evolution process (Figure 1). Trisomy 21 represents a “primary hit”, which alters hematopoiesis during embryonic development; acquisition of somatic *GATA1s* mutations represents a “secondary hit”, which promotes hematopoietic deregulation and emergence of TAM in DS newborns; additional mutations predominantly affecting chromatin and epigenetic regulators (e.g., the cohesin complex) and signaling mediators (e.g., Janus kinase 2, JAK2) represent a “tertiary hit”, which leads to ML-DS (Labuhn et al., 2019; Garnett et al., 2020; De Castro et al., 2021) (Figure 1). The detailed mechanism of how these events contribute to different stages of disease is still unclear. One of the studies showed that *GATA1s* mutations lead to TAM when introduced into trisomy 21 long-term hematopoietic stem cells (LT-HSCs), where a subset of chromosome 21 microRNAs (miRNAs) influences predisposition toward pre-leukemia initiation (Wagenblast et al., 2021). However, progression to ML-DS was independent of trisomy 21 in this study, but required synergy between mutations in *GATA1* and the cohesin genes, in particular cohesin subunit SA-2 (*STAG2*) knockout occurring in fetal or early postnatal but not adult HSCs (Wagenblast et al., 2021). Our review was motivated by these and other recent advances in the field that will likely open up new lines of research into ML-DS pathogenesis and targeted treatment development. We provide a comprehensive and up-to-date summary of molecular alterations in ML-DS, with an overriding aim to help guide future mechanistic studies into the pathogenesis of this disease. However, this is a rapidly advancing field, so despite our efforts this review may not be complete.

## Trisomy 21

Trisomy 21 is associated with defects in hematopoiesis and the immune system. Trisomy 21 fetuses have dysregulated development of megakaryocytic, erythroid and B-cell lineages (Laurent et al., 2020; De Castro et al., 2021). The mechanism through which an extra copy of chromosome 21 perturbs hematopoiesis and then how it cooperates with subsequent mutations to lead to TAM and ML-DS are still uncertain. Findings from a humanized model of pre-malignant and malignant stages of ML-DS demonstrated that trisomy 21 was necessary for pre-leukemia initiation but dispensable for leukemia progression (Wagenblast et al., 2021). The predominant current view is that DS-associated myeloproliferations result from deregulation of genes on human chromosome 21 (HSA21) estimated to contain 234 protein-coding genes (Antonarakis, 2017). These include genes critical for myeloid differentiation, such as ETS-related gene (*ERG*), ETS proto-oncogene 2 (*ETS2*), runt-related transcription factor 1 (*RUNX1*), dual specificity tyrosine phosphorylation regulated kinase 1A (*DYRK1A)*, regulator of calcineurin 1 (*RCAN1)*, chromatin assembly factor 1 subunit B (*CHAF1B*), high mobility group nucleosome binding domain 1 (*HMGN1*), SON DNA and RNA binding protein (*SON)*, and a subset of miRNAs (Table 1) (Laurent et al., 2020; Vukadin et al., 2021; Wagenblast et al., 2021). The encoded molecules belong to several functional classes, such as transcription factors, signaling effectors, epigenetic regulators, and miRNAs.

**TABLE 1:** List of HSA21 genes involved in myeloid proliferation associated with Down syndrome.

HSA21 genes	Classification	Function in hematopoiesis/leukemogenesis	References
*ERG*	Transcription factor	Causes megakaryoblastic expansion; involved in megakaryocytic leukemia; cooperates with GATA1s to drive TAM/ML-DS	Rainis et al. (2005); Salek-Ardakani et al. (2009); Stankiewicz and Crispino (2009); Carmichael et al. (2012); Stankiewicz and Crispino (2013); Banno et al. (2016)
*EST2*	Transcription factor	Regulates megakaryopoiesis; cooperates with GATA1s to drive TAM/ML-DS	Rainis et al. (2005); Ge et al. (2008); Stankiewicz and Crispino (2009); Banno et al. (2016)
*RUNX1*	Transcription factor	Involved in the pathogenesis of megakaryoblastic leukemia; causes abnormal megakaryocytic differentiation in cooperation with ERG, ETS2 and GATA1s; involved in TAM/ML-DS development	Elagib et al. (2003); Yanagida et al. (2005); Banno et al. (2016)
*BACH1*	Transcription factor	Inhibits megakaryocyte differentiation and platelet production	Toki et al. (2005)
*SON*	Transcription factor	Regulates hematopoiesis; represses megakaryocytic differentiation in megakaryoblastic leukemia	Belmonte et al. (2021); Vukadin et al. (2021)
*C21ORF66*	Transcription factor	Unknown	Reymond et al. (2001); Bourquin et al. (2006)
*GABPA*	Transcription factor	Regulates hematopoiesis and involved in CML development; role in TAM/ML-DS unknown	Yang et al. (2013); Manukjan et al. (2015)
*DYRK1A*	Signaling effector	Promotes TAM/ML-DS in human and murine models; co-operates with GATA1s to increase megakaryoblastic proliferation through NFAT inhibition	Malinge et al. (2012)
*RCAN1*	Signaling effector	Promotes megakaryopoiesis by inhibiting calcineurin-NFAT pathway	Zaslavsky et al. (2013)
*HMGN1*	Epigenetic modulator	Regulates myeloid differentiation; promotes leukemic stem cell activity by increasing H3K27 acetylation	Cabal-Hierro et al. (2020)
*CHAF1B*	Epigenetic modulator	Regulates hematopoiesis; impairs myeloid differentiation and promotes myeloid leukemia through binding of chromatin and interference with transcription factors such as CEBPA	Volk et al. (2018)
*miR-99a*	miRNA	Increases predisposition toward TAM but not ML-DS; has oncogenic function	Zhang et al. (2013); Si et al. (2016); Wagenblast et al. (2021)
*miR-125b*	miRNA	Increases predisposition toward TAM; regulates megakaryopoiesis; has oncogenic function; synergizes with GATA1s to induce megakaryoblastic leukemia	Klusmann et al. (2010); Alejo-Valle et al. (2021); Wagenblast et al. (2021)
*miR-155*	miRNA	Increases predisposition toward TAM but not ML-DS	Elton et al. (2010); Sas et al. (2020); Wagenblast et al. (2021)

### Transcription factors

The roles of ERG, ETS2 and RUNX1 in hematopoiesis and leukemogenesis have been thoroughly studied. ERG, an ETS transcription factor, is a megakaryocytic oncogene; its overexpression facilitates megakaryocytic expansion and promotes lymphoid and erythro-megakaryocytic leukemia *in vitro* and *in vivo* (Rainis et al., 2005; Salek-Ardakani et al., 2009; Stankiewicz and Crispino, 2009; Carmichael et al., 2012). Increased expression of ERG alone contributes to rapid onset of leukemia in mice (Salek-Ardakani et al., 2009). ERG strongly cooperates with the GATA1s mutated protein to immortalize hematopoietic and megakaryocytic progenitors *ex vivo* (Salek-Ardakani et al., 2009; Stankiewicz and Crispino, 2009). ERG and protein kinase B (PKB) also crosstalk, which alters GATA1 function (Stankiewicz and Crispino, 2013). Similar to ERG, ETS2 is an ETS transcription factor and a megakaryocytic oncogene (Ge et al., 2008). ETS2 promotes megakaryopoiesis and collaborates with GATA1s to immortalize hematopoietic progenitor cells (HPCs) (Rainis et al., 2005; Stankiewicz and Crispino, 2009). RUNX1 is a crucial transcription factor involved in the regulation of megakaryopoiesis, and its expression and cooperation with GATA1s facilitates megakaryocytic differentiation (Elagib et al., 2003). In 2005, overexpression of *RUNX1* was reported in bone marrow of ML-DS children (Langebrake et al., 2006). A subsequent report from 2006 showed *RUNX1* expression was lower than anticipated in ML-DS, while it was higher in megakaryoblasts from children with non-DS-AMKL (Bourquin et al., 2006). It appears that *SON*, another HSA21 gene, inhibits *RUNX1* expression (Vukadin et al., 2021), which may neutralize trisomy 21-related overdosage of *RUNX1* effects. Evidence from animal studies indicates that *RUNX1* overexpression in mice shortens the latency of leukemia development displaying enhanced frequency of megakaryoblastic leukemia, which supports that *RUNX1* overexpression is leukemogenic in ML-DS (Yanagida et al., 2005). Data from disease models using human induced pluripotent stem cells (iPSCs) and genome-editing technologies showed that an extra copy of *RUNX1* is essential for accelerating early hematopoiesis in the context of trisomy 21, leading to HPC expansion and increased myeloid differentiation (Banno et al., 2016). *RUNX1* expression level in trisomy 21 (*GATA1* wild type) iPSCs is increased by ∼1.8-fold compared with that in disomy 21 (*GATA1* wild type) iPSCs, which is slightly higher than the expected change in gene dosage (Banno et al., 2016). Abnormal megakaryocyte differentiation in TAM is accelerated by trisomy 21. Trisomy 21 up-regulates *GATA1s* expression leading to aberrant megakaryopoiesis, and the overdosage of *RUNX1*, *ETS2*, and *ERG* accelerates production of aberrantly differentiated cells (Banno et al., 2016). These observations highlight the importance of synergy between trisomy 21 and *GATA1s* in driving myeloid proliferation in DS children.

Other transcription factor encoding genes located on HSA21 are also highly expressed in ML-DS, including BTB domain and CNC homolog 1 (*BACH1*) (1.98-fold), *SON* (1.84-fold), chromosome 21 open reading frame 66 (*C21ORF66*) (1.64-fold) and GA-binding protein alpha chain (*GABPA*) (1.53-fold) (Bourquin et al., 2006). BACH1 acts as a transcriptional repressor of normal megakaryopoiesis and is likely a target of GATA1 and SON (Bourquin et al., 2006). Overexpression of *BACH1* causes maturation arrest of megakaryocytes resulting in marked peripheral thrombocytopenia (Toki et al., 2005). *SON* is a gene with homology to the proto-oncogene MYC family, and an RNA splicing factor regulating transcription of leukemia-associated genes. *SON* is indispensable for proper blood cell formation, as *SON* knockdown results in lower amounts of all myeloid cells and T cells (Belmonte et al., 2021). Megakaryocytic differentiation in AMKL is impaired by *SON* inhibiting expression of *RUNX1* and other megakaryocytic genes (Vukadin et al., 2021). *SON* also negatively regulates the expression of the AP-1 complex subunits JUN, JUNB and FOSB, which suggests that overexpression of *SON* could be pathogenic in ML-DS (Vukadin et al., 2021). *C21ORF66* is known as the GC-rich sequence DNA-binding factor candidate (Reymond et al., 2001), but its function is unknown. Further work is needed to elucidate the role of *C21ORF66* in hematopoiesis and leukemogenesis. *GABPA* has a known role in hematopoiesis (Yang et al., 2013). Deletion of *GABPA* leads to cell cycle arrest in hematopoietic stem cells (HSCs) and profound loss of HPCs (Yang et al., 2013). *GABPA* is necessary for chronic myeloid leukemia (CML) development through its regulation of protein kinase D2 (PRKD2) (Yang et al., 2013). *GABPA* expression positively correlates with the *BCR::ABL1/ABL1* ratio in cells from patients with CML, and influences imatinib sensitivity in leukemic cell lines (TKI-sensitive K-562 and TKI-resistant NALM-1) (Manukjan et al., 2015). However, the function of *GABPA* in the setting of trisomy 21 and *GATA1* mutations is not clear.

### Signaling effectors

DYRK1A belongs to the CMGC kinase group named after the initials of its subgroup members, including cyclin-dependent kinases, mitogen-activated protein kinases (MAPK), glycogen synthase kinases and CDK-like kinases. DYRK1A participates in various cellular functions through the phosphorylation of several substrates such as nuclear factor of activated T cells (NFAT) (Lindberg and Meijer, 2021). *DYRK1A* is a potent megakaryoblastic tumor-promoting gene, contributing to leukemogenesis in a mouse model containing 33 gene orthologs of HSA21, a *GATA1s* mutation, and a *MPL* mutation (Malinge et al., 2012). *DYRK1A* overexpression induces a marked megakaryoblastic proliferation through the suppression of NFAT in this model (Malinge et al., 2012). *RCAN1*, also known as Down syndrome critical region gene 1 (*DSCR1*), is an endogenous calcineurin inhibitor. Overexpression of *RCAN1* represses calcineurin-NFAT pathway, which leads to the expansion of megakaryocytes and their progenitors, and a high number of platelets (Zaslavsky et al., 2013). Both *DYRK1A* and *RCAN1* can down-regulate calcineurin-NFAT pathway, but little is known about how these signaling molecules collaborate with other HSA21 genes and *GATA1* mutations to initiate megakaryocytic neoplasia.

### Epigenetic modulators

HMGN1 is the chromatin accessibility regulator and a target of recurrent DNA copy gains in leukemia (Cabal-Hierro et al., 2020). *HMGN1* overexpression blocks myeloid differentiation, increases clonal progenitor expansion, enhances HSC activity and leukemic stem cell (LSC) activity in the presence of RUNX1::RUNX1T1 fusion oncoprotein (Cabal-Hierro et al., 2020). In addition, *HMGN1* up-regulation elevates *H3K27* acetylation, and in turn histone acetyltransferase CBP/p300 inhibition reverses the HMGN1-induced differentiation arrest. Another epigenetic modulator coded by a gene on HSA21 is CHAF1B, representing the p60 subunit of the chromatin assembly factor complex (Volk et al., 2018). CHAF1B is essential for normal hematopoiesis, whereas its overexpression promotes leukemia by binding chromatin at discrete sites and interfering with the occupancy of CCAAT enhancer binding protein alpha (CEBPA) (Volk et al., 2018). *CHAF1B* expression is higher in patient cells from ML-DS than those of non-DS-AMKL (Malinge et al., 2012). Reducing CHAF1B activity is sufficient to suppress leukemogenesis in mice without impairing normal hematopoiesis, suggesting CHAF1B is a potential therapeutic target (Volk et al., 2018). Overall, HMGN1 and CHAF1B block myeloid differentiation and promote leukemia growth in other contexts but their roles in the initiation of TAM and progression to ML-DS are not known.

### miRNAs

miRNAs, endogenous non-coding RNAs (∼23 nucleotides in length), target mRNA of protein-coding genes to regulate expression, through which they control a range of cellular processes, such as cell proliferation, apoptosis, hematopoiesis and tumorigenesis (Brás et al., 2018). A number of HSA21 miRNA genes are up-regulated in DS, including miR-155, miR-802, miR-125b-2, let-7c and miR-99a. Deregulated expression of miRNAs may contribute to a range of phenotypes in patients with DS, not only leukemia but also brain pathology, congenital heart defects, as well as low incidence of solid tumors in DS individuals (Brás et al., 2018). The miR-99a∼125b cluster, encoding let-7c, miR-99a and miR-125b, is highly expressed in TAM, ML-DS, and non-DS AMKL (Emmrich et al., 2014). The role of some HSA21 miRNAs in TAM/ML-DS pathogenesis has been partially revealed in recent years. (Alejo-Valle et al., 2021). *GATA1* mutations and miR-99a∼125b cluster interact to induce the block in megakaryocytic differentiation that leads to the expansion of megakaryocytic progenitors and AMKL in a mouse model (Alejo-Valle et al., 2021). Another study highlighted the role of three HSA21 miRNAs (miR-99a, miR-125b-2, and miR-155) in the development of TAM, but not ML-DS (Wagenblast et al., 2021). Co-expression of miR-99a, miR-125b-2, and miR-155 in normal fetal liver LT-HSCs recapitulates features of a trisomy 21-like hematopoietic state, while deletion of these miRNAs reduces the blast population in the presence of *GATA1s*. Nevertheless, in the mouse model of ML-DS with and without deletion of HSA21 miRNAs blast numbers are similar (Wagenblast et al., 2021). Other studies suggest that miR-99a plays an oncogenic role through increasing proliferation and colony forming ability, and decreasing apoptosis of hematopoietic progenitors (Zhang et al., 2013; Si et al., 2016). miR-125b-2 is a positive modulator of megakaryopoiesis and an oncogenic miRNA in ML-DS. miR-125b-2 up-regulation promotes proliferation and self-renewal of megakaryocytic and megakaryocytic/erythroid progenitors, while its down-regulation inhibits growth of ML-DS cells (Klusmann et al., 2010). Moreover, miR-125b-mediated repression of the megakaryocytic transcription factor AT-rich interactive domain-containing protein 3A (ARID3A) is a critical event in ML-DS pathogenesis (Alejo-Valle et al., 2021). In the context of miR-125b overexpression and *GATA1s* mutations, *ARID3A* is the main target of miR-125b. Down-regulation of *ARID3A* blocks megakaryocytic differentiation and subsequently AMKL, while restoring *ARID3A* expression reverses megakaryocytic differentiation arrest in AMKL patient-derived xenografts. This suggests that restoration of *ARID3A* could be a promising strategy to inhibit megakaryoblastic leukemia growth. miR-155, a known regulator of the immune system, is also a crucial player in TAM through targeting tumor necrosis factor (TNF) superfamily receptors; miR-155 expression increases 2-fold and 3-fold in DS fetal and adult cells, respectively (Elton et al., 2010; Sas et al., 2020). How miR-155-modulated TNF receptor expression promotes TAM/ML-DS remains unknown.

### Other effects of trisomy 21

Beyond the direct impact of HSA21 genes on myeloid proliferation, trisomy 21 also alters non-HSA21 gene expression through modulating genome organization (Letourneau et al., 2014; Liu et al., 2015; Ahlfors et al., 2019). Genome-wide studies showed that trisomy 21 has profound effects on DNA methylation in fetal and neonatal hematopoietic cells (Muskens et al., 2021). How these epigenetic changes influence TAM and ML-DS is not yet known. However, it has been shown that prior to the acquisition of *GATA1* mutations, trisomy 21 causes loss of DNA methylation at genes linked with the regulation of the cardiovascular, neurological, and endocrine organs. ML-DS has a unique epigenetic pattern characterized by gains of DNA methylation at genes correlated with hematopoiesis, cell proliferation, cell death, and cell cycle, which is distinct from other subtypes of pediatric AML, including non-DS-AMKL (Malinge et al., 2013). Significantly, TAM and ML-DS share the identical landscape of epigenetic changes (Malinge et al., 2013). Hence, it is possible that altered DNA methylation contributes towards development of TAM and ML-DS.

## *GATA1* mutations

Mutations in *GATA1* causing expression of its short isoform (*GATA1s*) are detected in nearly every case of TAM and ML-DS, implying mutated GATA1 deregulation plays a central role in TAM and ML-DS development (Wechsler et al., 2002; Panferova et al., 2021). The lack of detected *GATA1* mutations in ML-DS may be due to technical and sample limitations similar to those listed earlier for TAM. In addition, AMKL is associated with bone marrow fibrosis, which often impacts the quality and quantity of diagnostic bone marrow aspirate samples, in particular blast numbers. Because blasts are the cells that carry *GATA1s* in ML-DS, their paucity may limit detection. Similar to DS-like TAM, ML-DS-like leukaemia may arise in children without DS where *GATA1s* and trisomy 21 are somatically acquired in leukemic blasts (Ono et al., 2015; De Rooij et al., 2017; Panferova et al., 2021), or *GATA1s* mutations may be germline (Hasle et al., 2022). ML-DS-like leukemia is very rare but it shares multiple pathologic and clinical features with ML-DS, including good prognosis (De Rooij et al., 2017), emphasizing the importance of recognizing ML-DS-like leukemia in non-DS children.

GATA1 is encoded by the gene located on chromosome X and acts as a master transcription factor essential for the development of erythroid and megakaryocytic lineages (Pevny et al., 1991). More than 100 types of *GATA1* mutations have been reported in DS. These mutations are predominantly insertions, deletions, or duplications occurring in exon 2 or surrounding sequences. *GATA1* mutations create an early stop codon that results in an exclusive expression of a short isoform of GATA1 protein (referred to as GATA1s) that lacks the N-terminal activation domain. Rarely, mutations in exon 3 generate GATA1 proteins with internal deletions. GATA1s can bind DNA but fails to initiate transcription, leading to deregulation of many downstream target genes (Wechsler et al., 2002). The cellular stage in which the functional and molecular consequences of *GATA1s* begin in the embryo has been narrowed down to the erythro-megakaryocytic subpopulation of progenitors with the following immunophenotype: CD34^+^CD43^+^CD235-CD11b-CD71^+^CD41^+^ (Nishinaka-Arai et al., 2021). The identification of this cellular stage should assist further studies into the pathogenesis of both TAM and ML-DS.

GATA1s promotes megakaryocytic progenitor expansion and disrupts megakaryocytic and erythroid differentiation (Shimizu et al., 2009; Chlon et al., 2015; Banno et al., 2016; Juban et al., 2021). This appears to involve synergistic interactions with other leukemogenic molecules; for example, *GATA1s* increases expression of miRNA-486-5p, an erythroid oncogenic miRNA (Shaham et al., 2015). In the presence of trisomy 21, *GATA1s* mutations are sufficient to drive TAM, and these mutations become undetectable when TAM resolves (Shimizu et al., 2009). Evidence from a range of cellular and animal disease models confirmed that TAM is initiated by increased gene dosage from chromosome 21 acting in cooperation with *GATA1s*. *GATA1s* mutation alone disrupts differentiation of megakaryocytes and promotes expansion of myeloid and megakaryocytic progenitors, while production of aberrant megakaryoblasts is strengthened on the background of trisomy 21 (Banno et al., 2016; Juban et al., 2021; Matsuo et al., 2021). TAM requires the synergy between trisomy 21 and *GATA1s* but leukemic transformation may be independent of trisomy 21 (Wagenblast et al., 2021; Arkoun et al., 2022). In contrast, synergy between *GATA1s* and subsequent “tertiary” molecular alterations is critical for progression of TAM to ML-DS. Evidence from sequential longitudinal studies highlights that pre-leukemic and leukemic clones are truly related, due to the fact that identical *GATA1* mutations are found in paired TAM and ML-DS samples (Hitzler et al., 2003; Saida et al., 2013). Although most TAM clones disappear by the age of 3 months, some heterogeneous clones persist during remission, and these carry different leukemia-initiating potential (Saida et al., 2013). ML-DS can be derived from a minor clone with a distinct *GATA1s* in TAM, but novel clones can also arise and become dominant (Xu et al., 2006; Saida et al., 2013; Labuhn et al., 2019).

So far, there is no solid proof of whether the type of *GATA1* mutations, the level of *GATA1s* expression, or the size of dominant *GATA1s*-bearing clones can predict progression from TAM to ML-DS (Alford et al., 2011; Grimm et al., 2021). Kanezaki et al. pointed out that the type of *GATA1* mutations influences expression of the GATA1s protein, and these expression levels are inversely linked with the risk of progression to ML-DS (Kanezaki et al., 2010). Nonetheless, in the clinical setting, persistence of *GATA1s* mutations is the most important risk factor associated with progression to ML-DS, even in cases with high GATA1s protein levels (Massey et al., 2006; Pine et al., 2007). The features used in the clinic to predict TAM progression to ML-DS include detection of minimal residual disease by flow cytometry (blasts >0.1%), persistence of patient-specific *GATA1s* mutation beyond 12 weeks from the initial diagnosis, and the appearance of thrombocytopenia (platelet count less than 100×10^9^/L) (Klusmann et al., 2008; Flasinski et al., 2018).

## Tertiary alterations

It has become well accepted that evolution from TAM to ML-DS relies on the acquisition of tertiary somatic mutations and additional chromosomal structural aberrations in *GATA1s*-mutated cells. Tertiary mutations seen in ML-DS most commonly affect genes encoding the cohesin complex, JAK family kinases, and epigenetic regulators; other mutations occur in genes recurrently mutated in other types of AML, including fms-like tyrosine kinase 3 (*FLT3*) and *TP53* (Table 2) (Yoshida et al., 2013; Labuhn et al., 2019; Panferova et al., 2021). Patients with TAM usually harbor fewer tertiary mutations than those with ML-DS, at the average of 0.4 and 1.6 variants per sample respectively (Labuhn et al., 2019). Most TAM cases carry only *GATA1s*, while additional somatic mutations are rare. Even if present in TAM, “third hit” mutations appear to be non-functional and un-linked from pre-leukemia or leukemia phenotype (Labuhn et al., 2019). By way of illustration, no autonomous or cytokine-induced signaling was found for *JAK1*, *JAK2*, *JAK3* or *MPL* variants by dual-luciferase assays with a signal transducer and activator of transcription 5 (STAT5) reporter at the TAM stage (Labuhn et al., 2019). During leukemic transformation, two to five additional mutations are found in a murine model of ML-DS. The most frequently altered genes encode signaling pathways (34%), members of the cohesin complex or its associated components (28.5%), and epigenetic regulators (22%) (Labuhn et al., 2019). The authors suggest that ML-DS progression is influenced by the cooperation between activated signaling pathways and deregulated epigenetic processes in the context of trisomy 21 and *GATA1s*. For instance, a remarkable co-occurrence of variants in genes encoding tyrosine kinases (e.g., JAK2-3) and RAS proteins with variants in epigenetic regulators (e.g., enhancer of zeste 2, EZH2) or cohesin genes has been shown in ML-DS mouse models and ML-DS patients (Labuhn et al., 2019). No tertiary mutations were detected in approximately 15–25% of ML-DS patients in relatively large studies reported in the last few years (Labuhn et al., 2019; Panferova et al., 2021). However, it is possible that such mutations will be detected in the future using updated sequencing methodologies. Karyotypic changes other than trisomy 21 may also contribute to ML-DS because such alterations are rarely found at the TAM stage.

**TABLE 2:** Recurrent somatic mutations reported in myeloid leukemia associated with Down syndrome.

Class	Mutant genes	Frequency of mutations in various studies n (%)	Function in hematopoiesis/leukemogenesis; pathway to which it contributes	References
Cohesin complex and associated components	*CTCF*	16/141 (11.3); 10/49 (20.4); 5/44 (11.4)	Tumor suppressor; involved in chromatin organization, gene regulation, RNA splicing, myeloid cell growth and differentiation; contributes to leukemogenesis	Bell et al. (1999); Bell and Felsenfeld (2000); Torrano et al. (2005); Xu et al. (2007); Shukla et al. (2011); Yoshida et al. (2013); Zuin et al. (2014); Kim et al. (2017); Labuhn et al. (2019); Mujahed et al. (2020); Wang et al. (2020); Grimm et al. (2021); Panferova et al. (2021); Wagenblast et al. (2021)
	*NIPBL*	5/141 (3.5); 3/49 (6.1)	Cohesin regulator; regulates myeloid cell differentiation; contributes to leukemogenesis	Yoshida et al. (2013); Labuhn et al. (2019); Mazzola et al. (2019b); Mazzola et al. (2020); Wagenblast et al. (2021)
	*RAD21*	16/141 (11.3); 11/49 (22.4); 6/44 (13.6)	Cohesin subunit; regulates gene expression, epigenetic modulation, HSPC self-renewal and differentiation; contributes to leukemogenesis	Yoshida et al. (2013); Fisher et al. (2017); Labuhn et al. (2019); Bisaillon et al. (2020); Panferova et al. (2021); Wagenblast et al. (2021)
	*SMC1A*	9/141 (6.4); 2/49 (4.1); 1/44 (2.3)	Cohesin subunit; regulates gene expression, genome organization; contributes to leukemogenesis	Yoshida et al. (2013); Labuhn et al. (2019); Carico et al. (2021); Panferova et al. (2021); Wagenblast et al. (2021)
	*SMC3*	1/141 (0.7); 1/49 (2.0); 1/44 (2.3); 1/7 (14.3)	Cohesin ATPase subunit; contributes to hematopoietic failure and leukemogenesis	Nikolaev et al. (2013); Yoshida et al. (2013); Labuhn et al. (2019); Wang et al. (2019); Panferova et al. (2021); Rivas et al. (2021); Wagenblast et al. (2021); Arkoun et al. (2022)
	*STAG2*	19/141 (13.5); 9/49 (18.4); 4/44 (9.1)	Cohesin subunit; regulates gene expression, epigenetic modulation, HSPC self-renewal and differentiation; contributes to leukemogenesis	Yoshida et al. (2013); Labuhn et al. (2019); Nie et al. (2019); Viny et al. (2019); Ochi et al. (2020; Panferova et al. (2021); Wagenblast et al. (2021); Arkoun et al. (2022); Barwe et al. (2022)
Epigenetic regulators	*ASXL1*	1/49 (2.0); 1/44 (2.3)	Regulates histone modifications; impairs hematopoiesis; involved in leukemogenesis	Yoshida et al. (2013); Nagase et al. (2018); Panferova et al. (2021)
	*BCOR*	2/141 (1.4); 2/49 (4.1); 1/44 (2.3)	Transcription factor; PRC1 component; leads to myeloid progenitor expansion; regulates myeloid differentiation; contributes to leukemogenesis	Yoshida et al. (2013); Kelly et al. (2019); Labuhn et al. (2019); Panferova et al. (2021)
	*DNMT1*	1/44 (2.3)	Involved in DNA methylation; regulates hematopoiesis; contributes to leukemogenesis	Panferova et al. (2021); Chattopadhyaya and Ghosal ((2022)
	*DNMT3A*	1/49 (2.0)	Involved in DNA methylation; causes HSC expansion and impairs differentiation	Yoshida et al. (2013); Izzo et al. (2020)
	*EED*	1/141 (0.7)	PRC2 subunit; increases HSPC proliferation and impairs differentiation; contributes to leukemogenesis	Ikeda et al. (2016); Labuhn et al. (2019)
	*EP300*	1/141 (0.7)	Transcriptional cofactor; chromatin modifier; increases HSCs self-renewal and impairs differentiation; contributes to leukemogenesis	Labuhn et al. (2019); Man et al. (2021)
	*EZH2*	10/141 (7.1); 16/49 (32.7); 1/44 (2.3); 1/7 (14.3)	Tumor suppressor; PRC2 subunit; chromatin modifier; regulates histone modifications; inhibits megakaryocyte differentiation; contributes to leukemogenesis	Ntziachristos et al. (2012); Nikolaev et al. (2013); Yoshida et al. (2013); Labuhn et al. (2019); Mazzi et al. (2021); Panferova et al. (2021)
	*KANSL1*	17/141 (12.1); 3/49 (6.1)	Regulates histone acetylation; contributes to leukemogenesis	Yoshida et al. (2013); Labuhn et al. (2019); Wagenblast et al. (2021)
	*KAT6A*	1/44 (2.3)	Oncogene; regulates histone acetylation; impairs myeloid differentiation; contributes to leukemogenesis	Panferova et al. (2021); Yan et al. (2021)
	*KDM6A*	1/141 (0.7)	Regulates histone modifications; regulates hematopoiesis; contributes to leukemogenesis	Labuhn et al. (2019); Tian et al. (2021)
	*KMT2C*	1/141 (0.7)	Tumor suppressor; regulates histone modifications; involved in myelopoiesis; contributes to leukemogenesis	Labuhn et al. (2019); Maurya et al. (2021)
	*NAT6*	1/141 (0.7)	Regulates actin acetylation	Labuhn et al. (2019); Muffels et al. (2021)
	*SUZ12*	9/141 (6.4); 1/49 (2.0); 1/44 (2.3)	PRC2 subunit; tumor suppressor; chromatin modifier; regulates histone modifications and HSCs activity; contributes to leukemogenesis	Majewski et al. (2008); Ntziachristos et al. (2012); Yoshida et al. (2013); Labuhn et al. (2019); Panferova et al. (2021)
	*TET2*	2/141 (1.4); 3/44 (6.8)	Involved in DNA methylation; causes HSC expansion and impairs differentiation	Labuhn et al. (2019); Izzo et al. (2020); Panferova et al. (2021)
Tyrosine kinases	*FLT3*	1/44 (2.3); 1/7 (14.3); 2/7 (28.6)	PI3K-PKB; MAPK; regulates hematopoiesis; contributes to leukemogenesis	Gilliland and Griffin (2002); Malinge et al. (2008); Grafone et al. (2012); Nikolaev et al. (2013); Panferova et al. (2021)
	*GNB1*	1/141 (0.7)	PI3K-PKB; MAPK	Zimmermannova et al. (2017); Labuhn et al. (2019)
	*JAK1*	6/141 (4.3); 2/49 (4.1); 3/44 (6.8); 1/7 (14.3)	JAK-STAT; regulates hematopoiesis; contributes to leukemogenesis	Nikolaev et al. (2013); Yoshida et al. (2013); Labuhn et al. (2019); Fasouli and Katsantoni (2021); Panferova et al. (2021)
	*JAK2*	14/141 (9.9); 4/49 (8.2); 4/44 (9.1); 1/7 (14.3)	JAK-STAT; regulates hematopoiesis; contributes to leukemogenesis	Malinge et al. (2008); Yoshida et al. (2013); Labuhn et al. (2019); Fasouli and Katsantoni (2021); Panferova et al. (2021)
	*JAK3*	19/141 (13.5); 6/49 (12.2); 12/44 (27.3); 1/7 (14.3); 1/11 (9.1); 1/3 (33.3); 1/14 (7.1)	JAK-STAT; regulates hematopoiesis; contributes to leukemogenesis	Walters et al. (2006); Kiyoi et al. (2007); Klusmann et al. (2007); Malinge et al. (2008); Yoshida et al. (2013); Labuhn et al. (2019); Fasouli and Katsantoni (2021); Panferova et al. (2021)
	*KIT*	2/141 (1.4)	Kit signaling; regulates hematopoiesis; contributes to leukemogenesis	Stankov et al. (2014); Labuhn et al. (2019)
	*MPL*	10/141 (7.1); 3/49 (6.1); 1/44 (2.3)	MPL signaling; JAK-STAT; regulates megakaryopoiesis; contributes to leukemogenesis	Yoshida et al. (2013); Labuhn et al. (2019); Loscocco et al. (2020); Nakamura-Ishizu and Suda (2020); Panferova et al. (2021); Arkoun et al. (2022)
	*NTRK3*	1/44 (2.3)	Oncogene; JAK-STAT; PI3K/PKB; MAPK; contributes to leukemogenesis	Joshi et al. (2020); Panferova et al. (2021)
	*PI3KC2A*	1/7 (14.3)	PI3K member; insulin signaling; human cytomegalovirus virions production	Nikolaev et al. (2013); Polachek et al. (2016); Zhuo et al. (2017)
	*PTEN*	1/141 (0.7)	Tumor suppressor; PI3K/PKB/mTOR; regulates hematopoiesis; contributes to leukemogenesis	Labuhn et al. (2019); Wu et al. (2020)
	*PTPRD*	1/141 (0.7)	Tumor suppressor; contributes to leukemogenesis	Song et al. (2016); Labuhn et al. (2019)
	*SH2B3*	4/141 (2.8); 4/49 (8.2); 2/44 (4.5)	JAK-STAT, PKB, MAPK; regulates thrombopoiesis; contributes to leukemogenesis	Yoshida et al. (2013); Maslah et al. (2017); Labuhn et al. (2019); Panferova et al. (2021)
**RAS**	*KRAS*	7/141 (5.0); 4/49 (8.2); 2/44 (4.5)	Oncogene; RAS signaling; KRAS/RAC1/ROS/NLRP3/IL-1β; regulates hematopoiesis; contributes to leukemogenesis	Yoshida et al. (2013); Sasine et al. (2018); Labuhn et al. (2019); Hamarsheh et al. (2020); Panferova et al. (2021)
	*NF1*	4/141 (2.8)	Tumor suppressor; RAS signaling; regulates hematopoiesis; contributes to leukemogenesis	Zhang et al. (2001); Labuhn et al. (2019); Vara et al. (2020)
	*NRAS*	6/141 (4.3); 4/49 (8.2); 4/44 (9.1)	Oncogene; RAS signaling; regulates hematopoiesis; contributes to leukemogenesis	Yoshida et al. (2013); Gu et al. (2019); Labuhn et al. (2019); Shi et al. (2019); Panferova et al. (2021)
	*PTPN11*	1/49 (2.0)	Oncogene; RAS signaling; regulates hematopoiesis; contributes to leukemogenesis	Yoshida et al. (2013); Pandey et al. (2017)
Transcription factors	*CREBBP*	1/141 (0.7)	Tumor suppressor; transcriptional coactivator; lysine acetyltransferase enzyme; regulates hematopoiesis; contributes to leukemogenesis	Labuhn et al. (2019); Zhang et al. (2021)
	*GATA2*	1/44 (2.3)	Transcription factor; regulates early hematopoiesis (HSPC generation and function); contributes to leukemogenesis	Fujiwara (2017); Soukup and Bresnick (2020); Panferova et al. (2021)
	*MYC*	1/141 (0.7); 1/44 (2.3)	Oncogene; transcription factor; regulates hematopoiesis; contributes to leukemogenesis	Labuhn et al. (2019); Benetatos et al. (2020); Panferova et al. (2021)
	*RUNX1*	3/141 (2.1)	Transcription factor; master-regulator of hematopoiesis; regulates megakaryopoiesis; contributes to leukemogenesis	Elagib et al. (2003); Yanagida et al. (2005); Banno et al. (2016); Gonzales et al. (2021); Panferova et al. (2021)
	*TAL1*	1/44 (2.3)	Oncogene; transcription factor; regulates HSC; contributes to leukemogenesis	Panferova et al. (2021); Thoms et al. (2021)
	*TP53*	5/141 (3.5); 3/49 (6.1); 2/44 (4.5); 2/11 (18.2)	Tumor suppressor; transcription factor; regulates hematopoiesis; contributes to leukemogenesis	Kiyoi et al. (2007); Yoshida et al. (2013); Labuhn et al. (2019); George et al. (2021); Panferova et al. (2021)
	*WT1*	1/141 (0.7); 2/49 (4.1); 1/44 (2.3)	Transcriptional activator or repressor; regulates hematopoiesis; contributes to leukemogenesis	Yoshida et al. (2013); Labuhn et al. (2019); Panferova et al. (2021); El Hussein et al. (2022)
Others	*CARD11*	1/44 (2.3)	Oncogene; TCR and BCR signaling; regulates hematopoiesis; contributes to leukemogenesis	Lu et al. (2021); Panferova et al. (2021)
	*CHEK2*	2/44 (4.5)	DNA damage response gene; contributes to leukemogenesis	Bazinet et al. (2021); Panferova et al. (2021); Singh et al. (2022)
	*CSF2RB*	7/141 (5.0)	Oncogene; JAK-STAT; PI3K-PKB- mTOR; MEK/ERK; regulates megakaryocytic proliferation and differentiation; contributes to leukemogenesis	Labuhn et al. (2019)
	*CSF3R*	1/44 (2.3)	Oncogene; JAK-STAT; regulates granulocyte progenitor differentiation; contributes to leukemogenesis	Maxson and Tyner (2017); Panferova et al. (2021)
	*DCAF7*	1/141 (0.7); 2/49 (4.1)	Scaffold protein or adaptor protein; interacts with ERCC1-XPF, DYRK1A, DYRK1B, MEKK1, and HIPK2	Yoshida et al. (2013); Yousefelahiyeh et al. (2018); Kawara et al. (2019); Labuhn et al. (2019)
	*DLEC1*	1/7 (14.3)	Tumor suppressor	Nikolaev et al. (2013); Hong et al. (2016)
	*DHX29*	1/7 (14.3)	RNA helicase; RNA co-sensor for anti- encephalomyocarditis virus immunity; regulates translation initiation	Nikolaev et al. (2013); Sweeney et al. (2021); Zhou et al. (2021)
	*ETNK1*	1/44 (2.3)	Kinase; involved in ethanolamine phosphorylation, ROS production, and DNA damage	Fontana et al. (2020); Panferova et al. (2021)
	*PML*	1/44 (2.3)	Tumor suppressor; regulates hematopoiesis; contributes to leukemogenesis	Haupt et al. (2013); Panferova et al. (2021)
	*POLE*	1/7 (14.3)	DNA replication; cancer-predisposing gene	Nikolaev et al. (2013); Magrin et al. (2021)
	*PRPF40B*	1/44 (2.3)	RNA splicing machinery; contributes to leukemogenesis	Lorenzini et al. (2019); Panferova et al. (2021)
	*SF3B1*	3/141 (2.1)	RNA splicing machinery; contributes to leukemogenesis	Labuhn et al. (2019); Van Der Werf et al. (2021)
	*SRSF2*	12/141 (8.5); 1/49 (2.0)	RNA splicing machinery; contributes to leukemogenesis	Hama et al. (2008); Yoshida et al. (2013); Labuhn et al. (2019); Todisco et al. (2021)
	*WRN*	1/44 (2.3)	Helicases; DNA replication and repair machinery; contributes to leukemogenesis	Moles et al. (2016); Panferova et al. (2021)

### Mutations in the cohesin complex and related components

Cohesin is a multi-subunit complex composed of three main structural proteins (structural maintenance of chromosomes protein 1A (SMC1A), structural maintenance of chromosomes protein 3 (SMC3), and double-strand-break repair protein Rad21 homolog (RAD21)), which bind to either cohesin subunit SA-1 (STAG1) or cohesin subunit SA-2 (STAG2) proteins. Cohesin complex is a ring-shaped structure that surrounds chromosomal DNA and controls its functions, including sister chromatid cohesion, chromatin remodeling, transcriptional regulation, and DNA damage repair (Jann and Tothova, 2021). Nipped-B-like protein (NIPBL) is involved in cohesin loading to chromatin, translocating cohesin along chromatin fibers, and regulating cohesin after loading (Garcia et al., 2021). Cohesin core subunits and its modulators (including STAG2, RAD21, SMC1A, SMC3 and NIPBL) are recurrently mutated in myeloid malignancies. Cohesin mutations are highly prevalent in ML-DS, where they occur in nearly half of patients (Yoshida et al., 2013). *STAG2* and *RAD21* have the higher mutation frequency than *SMC1*, *SMC3* and *NIPBL*, with approximately 9.1–18.4% and 11.3–22.4% in ML-DS cases respectively (Nikolaev et al., 2013; Yoshida et al., 2013; Labuhn et al., 2019; Panferova et al., 2021). Each of these mutations results in loss-of-function of the molecules, and in cooperation with *GATA1s* and trisomy 21 each can drive leukemic transformation.

Recent genetic modifications of human iPSC lines derived from DS tissue greatly assisted examination of the cooperativity between *GATA1s* and cohesin mutations in ML-DS. The clustered regularly interspaced short palindromic repeats (CRISPR)/Cas9 system was used to introduce *GATA1s* and *STAG2* mutations into iPSCs in a sequential manner (Barwe et al., 2020; Barwe et al., 2022). *GATA1s* and *STAG2* knockout cooperatively increased the megakaryocytic population and induced the ML-DS immunophenotype (Barwe et al., 2022). In another study, trisomic 21 iPSC line (Chou et al., 2012) was edited to introduce *GATA1s* followed by heterozygous inactivation of *SMC3* (*SMC3* ^+/−^) and then, introduction of a gain-of-function *MPL* mutation (MPLW515K) (Arkoun et al., 2022). It was found that *GATA1s* impaired megakaryocyte differentiation and that *SMC3* ^+/−^ enhanced this effect independent of trisomy 21. MPLW515K further increased the megakaryocyte output in this model, including through the induction of growth factor independence. Low expression of *NFE2* was critical for the induction of megakaryocyte dysplasia by *GATA1s* (Arkoun et al., 2022). These novel iPSC-based models are likely to rapidly advance our understanding of ML-DS pathogenesis, and assist therapy development.

We include a brief description of the relevant cohesin elements to highlight their roles in haematopoiesis. *STAG2*, located on the chromosome X, is the most frequently mutated cohesin gene in human cancer (Viny et al., 2019). *STAG2* deletion in hematopoietic stem and progenitor cells results in abnormal hematopoietic function, increased self-renewal, and impaired differentiation (Viny et al., 2019). *STAG2* loss-of-function decreases cell growth and proliferation, increases cell invasion and metastasis, enhances chemo-resistance, regulates the expression of many immune-related genes, and interplays with *RUNX1* deficiency to perturb chromatin looping (Nie et al., 2019; Ochi et al., 2020). Likewise, *RAD21* loss-of-function confers enhanced hematopoietic self-renewal and impaired cell differentiation (Fisher et al., 2017; Bisaillon et al., 2020). RAD21 is a regulator of gene expression and epigenetic modulation. For example, RAD21 regulates expression of *RUNX1* and methylation of Homeobox a7/Homeobox a9 (Fisher et al., 2017; Bisaillon et al., 2020). NIPBL also regulates *RUNX1* expression, thus its loss-of-function impairs *RUNX1* expression and consequently, hematopoiesis (Mazzola et al., 2020). NIPBL interplaying with nucleophosmin 1 (NPM1) regulates myeloid differentiation through the WNT (Wingless/Integrated) pathway; the disruption of these interactions has been implicated in leukemogenesis (Mazzola et al., 2019a). SMC3 is the cohesin ATPase subunit, with its dosage controlling embryogenesis and hematopoiesis (Wang et al., 2019; Rivas et al., 2021). Homozygous deletion of *SMC3* in mice results in embryonic lethality and the hematopoietic failure (Wang et al., 2019). In comparison, heterozygous *SMC3* deletion leads to developmental defects (e.g., abnormal craniofacial morphology), germinal center hyperplasia with increased B-cell proliferation and increased risk of B-cell lymphoma development (Rivas et al., 2021). SMC1A-R586W mutation is known to interfere with cohesin localization and cohesin-mediated DNA loop interaction in AML cells. This mutation confers wide changes to gene expression and genome organization when engineered into murine embryonic stem cells (Carico et al., 2021). Finally, CCCTC-binding factor (CTCF) is a tumor suppressor and involved in many cellular processes, with approximately 11.3–20.4% of ML-DS cases harboring *CTCF* gene mutations (Yoshida et al., 2013; Labuhn et al., 2019; Panferova et al., 2021). CTCF interacts with the cohesin complex to control genome architecture and gene expression (Zuin et al., 2014; Wang et al., 2020). CTCF and cohesins are known to assist formation of DNA loops, however depletion of either the cohesin complex or CTCF has differential effects on chromatin organization and gene expression in human HEK293T cells (Zuin et al., 2014). Deletion of cohesin caused a general loss of local chromatin interactions but the topological domains remained intact. In contrast, depletion of CTCF both reduced and increased interdomain interactions and distinct groups of genes became dysregulated. Apart from its interplay with cohesins, CTCF is a highly conserved transcription factor implicated in transcriptional activation and repression, insulation, formation of chromatin barrier, gene imprinting, X-chromosome inactivation, and RNA splicing (Bell et al., 1999; Bell and Felsenfeld, 2000; Xu et al., 2007; Shukla et al., 2011; Wang et al., 2020). CTCF is also involved in maintaining genomic methylation patterns through the control of poly (ADP-ribose) polymerase 1 (PARP1) and the activity of DNA methyltransferase 1 (DNMT1) (Zampieri et al., 2012). *CTCF* haploinsufficiency correlates with altered patterns of DNA methylation and predisposes to cancer in mice (Kemp et al., 2014). CTCF is a critical factor in the control of hematopoiesis and leukemogenesis (Torrano et al., 2005; Kim et al., 2017; Mujahed et al., 2020). In adult mice, conditional *CTCF* deletion causes an acute loss of HSCs, severe bone marrow failure and increased mortality, highlighting CTCF requirement for the maintenance of the HSC pool (Kim et al., 2017). Abnormal CTCF expression reduces growth and enhances differentiation of the erythroid lineage by down-regulating MYC (Torrano et al., 2005). In AML, CTCF binding was shown to be elevated, compared with normal bone marrow, with increased CTCF binding in promoter regions linked with DNA hypomethylation and increased target gene expression (Mujahed et al., 2020). However, the combination of *CTCF* loss-of-function with *GATA1s* and trisomy 21 is unable to drive leukemic transformation, indicating additional events are required (Wagenblast et al., 2021). Collectively, the cohesin complex and CTCF are involved in ML-DS pathogenesis, but the exact roles of these molecules need further elucidation.

### Mutations in signaling pathways

Most mutations affecting signaling pathways occur in genes encoding JAK regulators, *MPL* and *KIT* (CD117) collectively reported in 48% of ML-DS cases (Labuhn et al., 2019). *JAK2* and *JAK3* are more frequently mutated (9.9 and 13.5%) than *JAK1*, *MPL* and *KIT* (4.3, 7.1 and 1.4%) (Labuhn et al., 2019). *JAK1-3* variants are identified in both ML-DS and TAM samples, however, gain-of-function mutations are only detected in ML-DS, highlighting that aberrant activation of JAK-STAT signaling is important for transition to leukemia (Labuhn et al., 2019). The JAK family of tyrosine kinases (JAK1-3 and tyrosine kinase 2, TYK2) are pivotal mediators of growth factor and cytokine signaling, including downstream of thrombopoietin (TPO) and granulocyte-macrophage colony-stimulating factor (GM-CSF) (De Castro et al., 2021; Moser et al., 2021). JAK1, JAK2 and TYK2, are ubiquitously expressed, whilst JAK3 is predominantly expressed in lymphoid and myeloid cells. *JAK3* mutations are more common than of other members of JAK family in ML-DS (Labuhn et al., 2019). Under physiological conditions, JAK-STAT signaling is tightly controlled and involved in a wide range of fundamental biological processes, including cell proliferation, differentiation, apoptosis, inflammation, and blood production (Park et al., 2016; De Castro et al., 2021; Moser et al., 2021). Normal megakaryopoiesis requires TPO-mediated STAT5 activation. Unphosphorylated STAT5 represses megakaryocytic transcriptional program and inhibits megakaryocytic differentiation by competing with ERG for CTCF binding, which can be reversed by TPO-mediated activation of STAT5 (Park et al., 2016). MPL, a receptor of TPO, is also frequently mutated in ML-DS, which contributes to leukemia development. In the presence of trisomy 21 and GATA1s, MPL W515L causes rapid and lethal leukemia in mice (Malinge et al., 2012). Recently, colony stimulating factor 2 receptor subunit beta (CSF2RB) A455D variant was reported in almost 5% of ML-DS children. This variant is mutually exclusive with mutated *JAK1-3*, *MPL* or *RAS* genes, and causes ligand-independent STAT5 activation promoting cytokine-independent cell growth (Labuhn et al., 2019). Upon introduction of the CSF2RB A455D mutant into hematopoietic stem and progenitor cells (HSPCs), megakaryocytic and erythroid proliferation is enhanced, and terminal megakaryocytic maturation is blocked (Labuhn et al., 2019). These alterations are alleviated by the JAK1/2 inhibitor ruxolitinib, emphasizing that aberrant JAK-STAT signaling participates in the CSF2RB A455D-driven leukemogenesis (Labuhn et al., 2019). In addition, CSF2RB binding to FLT3-ITD is found in other AML cell lines and patient cells where CSF2RB deletion decreases STAT5 phosphorylation, inhibits leukemic cell proliferation, and sensitizes cells to FLT3 inhibition (Charlet et al., 2021). These findings demonstrate that CSF2RB is critical for FLT3-ITD-dependent oncogenic signaling and transformation, but its role in ML-DS requires further study.

Mutations in *RAS* (Rat sarcoma virus) gene family members, such as *KRAS*, *NF1*, *NRAS*, and *PTPN11,* are found in 14% of ML-DS samples (Labuhn et al., 2019). *NRAS* and *KRAS* variants are the most common accounting for 4.5–8.2% and 4.3–9.1% of ML-DS cases respectively (Yoshida et al., 2013; Labuhn et al., 2019; Panferova et al., 2021). Ras belongs to the small GTPase family that binds to guanosine triphosphate (GTP) and hydrolyses it to guanosine diphosphate (GDP), with three distinct isoforms NRas, KRas, and HRas (Padmakumar et al., 2021). Ras is located on the inner surface of the plasma membrane, and acts as a binary molecular switch. Ras can transmit extracellular signals to the nucleus, and cycles between the inactive GDP-bound state and the active GTP-bound state (Zafar et al., 2021). Mutations fix RAS-GTPase proteins in an active GTP-bound state, resulting in constitutive activation of MAPK and PI3K (phosphoinositide 3-kinases) signaling. Consequently, uncontrolled cell proliferation and survival occur in mutated cells. In mouse models, clonal NRAS/KRAS activation increases cell growth, proliferation, and colony formation through a lysine methyltransferase 2A (KMT2A)- polo like kinase 1 (PLK1) axis (Carr et al., 2021). Mutations in *RAS* have a role in TAM progression to ML-DS, but it is not fully understood how these mutations cooperate with trisomy 21, *GATA1s* and other mutations in cohesins or epigenetic modulators.

### Mutations in epigenetic regulators

Loss-of-function mutations in epigenetic regulators are emerging as critical contributors to ML-DS progression. Such mutations are reported in approximately 36–45% of ML-DS samples and affect a range of regulators, including additional sex combs-like 1 (*ASXL1*), BCL6 corepressor (*BCOR*), *DNMT1*, *DNMT3A*, embryonic ectoderm development (*EED*), E1A binding protein P300 (*EP300*), *EZH2*, KAT8 regulatory NSL complex subunit 1 (*KANSL1*), lysine demethylase 6A (*KDM6A*), lysine methyltransferase 2C (*KMT2C*), N-acetyltransferase 6 (*NAT6*), *SUZ12*, and tet methylcytosine dioxygenase 2 (*TET2*) (Nikolaev et al., 2013; Yoshida et al., 2013; Labuhn et al., 2019; Panferova et al., 2021). Mutations in *KANSL1*, *EZH2* and *SUZ12* were seen at the highest frequency, in 6.1–12.1%, 2.3–32.7% and 2–6.4% of ML-DS cases respectively (Nikolaev et al., 2013; Yoshida et al., 2013; Labuhn et al., 2019; Panferova et al., 2021). *KANSL1* is essential for the activity of the histone acetylation complex, which takes part in the acetylation of histone H4 lysine 16 and eventually leads to transcriptional activation. Loss-of-function mutations in *KANSL1* are detected in both ML-DS and non-DS-AMKL (Yoshida et al., 2013; Labuhn et al., 2019). *KANSL1* mutations combined with trisomy 21 and *GATA1s* drive leukemic engraftment in mice (Wagenblast et al., 2021). EZH2 forms polycomb repressive complex 2 (PRC2) together with SUZ12, EED and RB binding protein 4 (RBBP4). PRC2 is mainly responsible for the methylation of lysine 27 in the tail of histone H3 family proteins (H3K27me3), which subsequently silences its target gene expression. Thus, EZH2 is a transcriptional repressor with methyltransferase activity, whereas SUZ12 is essential for the structural integrity of PRC2 and the facilitation of chromatin binding (Chen et al., 2018; Zeisig and So, 2021). EZH2 is unable to perform this enzymatic function alone, and the interplay with EED and SUZ12 enables PRC2 function (Chen et al., 2018; Zeisig and So, 2021). In megakaryopoiesis, EZH2 inhibition accelerates megakaryocytic differentiation and blocks megakaryocytic proliferation (Mazzi et al., 2021). EZH2 and SUZ12 act as tumor suppressors; mutations in either gene lead to loss-of-function of PRC2 core subunits and a deficit of H3K27me3 (Ntziachristos et al., 2012). Murine ML-DS leukemia models and ML-DS patients show loss-of-function mutations in EZH2 and other PRC2 members, supporting the PRC2 role in transition from TAM to ML-DS (Labuhn et al., 2019). Although the importance of mutated epigenetic modifiers in ML-DS has been recognized, their pathologic functions and clinical impact remain unclear.

### Chromosomal abnormalities

Beyond trisomy 21, additional cytogenetic changes are observed in the majority of children with ML-DS, but rarely in TAM (Malinge et al., 2008; Labuhn et al., 2019). Therefore, these changes could play a role in the development of ML-DS. Cytogenetic changes reported in ML-DS include gains and losses of whole chromosomes or their arms, or chromosomal rearrangements (Table 3). Common chromosomal gains are trisomies: +2, +8, +11, +13, +14, +19, +22, or tetrasomies: +14, +21. Chromosomal losses include monosomies: −1, −3, −4, −5, −7, −9, −16, and −18. Other aberrations include: add(1q), add(6p), add (6q), add (7q), add(8p), add(11q), add (16q), add (19p); or deletions: del(5p), del(5q), del(6q), del(7p), del(7q), del(11p), del(13q), del(16q), del(17p), del(17q), and del(22q). The most common structural abnormalities are del(7p)/del(7q)/−7, del(16q), trisomy 8, and tetrasomy 21. However, none of these changes offer clear insights into the molecular pathogenesis of ML-DS, and their prognostic impact is also largely unknown (Forestier et al., 2008; De Souza et al., 2017; Labuhn et al., 2019). One recent study points out that +8 can be associated with inferior event-free survival in ML-DS (Uffmann et al., 2017). More work is required to elucidate the pathogenetic role and clinical impact of chromosomal abnormalities in ML-DS.

**TABLE 3:** Chromosomal abnormalities reported in myeloid leukemia associated with Down syndrome.

Class	Cytogenetic alteration	Frequency of alterations in various studies n (%)	References
Whole chromosome gain	Trisomy 2	1/141 (0.7)	Labuhn et al. (2019)
	Trisomy 8	9/141 (6.4); 1/7 (14.3); 4/24 (16.7)	Hama et al. (2008); Malinge et al. (2008); Labuhn et l. (2019)
	Trisomy 11	1/141 (0.7); 2/24 (8.3)	Hama et al. (2008); Labuhn et al. (2019)
	Trisomy 13	3/141 (2.1)	Labuhn et al. (2019)
	Trisomy 14	3/141 (2.1)	Labuhn et al. (2019)
	Tetrasomy 14	1/7 (14.3)	Nikolaev et al. (2013)
	Trisomy 19	2/141 (1.4); 1/24 (4.2)	Hama et al. (2008); Labuhn et al. (2019)
	Tetrasomy 21	9/141 (6.4); 1/7 (14.3); 1/24 (4.2); 1/7 (14.3)	Hama et al. (2008); Malinge et al. (2008); Nikolaev et al. (2013); Labuhn et al. (2019)
	Trisomy 22	1/141 (0.7); 1/24 (4.2)	Hama et al. (2008); Labuhn et al. (2019)
Chromosomal arm gain	add(1q)	4/141 (2.8)	Labuhn et al. (2019)
	add(5p)	1/24 (4.2)	Hama et al. (2008)
	add(5q)	1/24 (4.2)	Hama et al. (2008)
	add(6p)	1/141 (0.7)	Labuhn et al. (2019)
	add(6q)	1/141 (0.7)	Labuhn et al. (2019)
	add(7p)	2/24 (8.3)	Hama et al. (2008)
	add(7q)	2/141 (1.4)	Labuhn et al. (2019)
	add(8p)	1/141 (0.7)	Labuhn et al. (2019)
	add(11q)	1/141 (0.7)	Labuhn et al. (2019)
	add(16q)	2/141 (1.4)	Labuhn et al. (2019)
	add(19p)	1/141 (0.7); 1/24 (4.2)	Hama et al. (2008); Labuhn et al. (2019)
	add(22q)	1/24 (4.2)	Hama et al. (2008)
Whole chromosome loss	−1	1/24 (4.2)	Hama et al. (2008)
	−3	1/24 (4.2)	Hama et al. (2008)
	−4	1/7 (14.3)	Malinge et al. (2008)
	−5	1/24 (4.2)	Hama et al. (2008)
	−7	5/24 (20.8)	Hama et al. (2008)
	−9	1/24 (4.2); 1/7 (14.3)	Hama et al. (2008); Malinge et al. (2008)
	−16	1/7 (14.3)	Malinge et al. (2008)
	−18	1/24 (4.2)	Hama et al. (2008)
Chromosomal arm loss	del(5p)	1/141 (0.7)	Labuhn et al. (2019)
	del(5q)	3/141 (2.1); 1/7 (14.3)	Nikolaev et al. (2013); Labuhn et al. (2019)
	del(6q)	2/141 (1.4); 1/7 (14.3); 1/24 (4.2)	Hama et al. (2008); Nikolaev et al. (2013); Labuhn et al. (2019)
	del(7p)	5/141 (3.5);	Labuhn et al. (2019)
	del(7q)	2/141 (1.4); 1/24 (4.2)	Hama et al. (2008); Labuhn et al. (2019)
	del(8q)	1/7 (14.3)	Nikolaev et al. (2013)
	del(11p)	2/141 (1.4); 1/24 (4.2)	Hama et al. (2008); Labuhn et al. (2019)
	del(11q)	1/24 (4.2)	Hama et al. (2008)
	del(13q)	2/141 (1.4)	Labuhn et al. (2019)
	del(16q)	6/141 (4.3)	Labuhn et al. (2019)
	del(17p)	3/141 (2.1)	Labuhn et al. (2019)
	del(17q)	3/141 (2.1)	Labuhn et al. (2019)
	del(20q)	1/24 (4.2)	Hama et al. (2008)
	del(22q)	1/141 (0.7)	Labuhn et al. (2019)
Other changes	+der(1)t(1; ?)	1/24 (4.2)	Hama et al. (2008)
	der(3)t(3;3) (p25;p10)	1/24 (4.2)	Hama et al. (2008)
	+der(5)t(5;7)	1/24 (4.2)	Hama et al. (2008)
	der(7)t(1;7) (q23;q36)	1/24 (4.2)	Hama et al. (2008)
	der(14) t(1;14) (q24∼25;p11)	1/141 (0.7)	Labuhn et al. (2019)
	der(17)t(1;17) (q25;q25)	1/24 (4.2)	Hama et al. (2008)
	der(21) (qter- > q22.1::p11.2- > qter)	1/141 (0.7)	Labuhn et al. (2019)
	der(X)t(X;1) (q28;q25)	1/24 (4.2)	Hama et al. (2008)
	inv (9) (p11;q12)	1/7 (14.3)	Malinge et al. (2008)
	isochromosome (7q)	1/7 (14.3); 1/24 (4.2)	Hama et al. (2008); Nikolaev et al. (2013)
	random aberrations	2/7 (28.6)	Malinge et al. (2008)
	t(3;17) (q25;q25)	1/141 (0.7)	Hama et al. (2008)
	t(5;12) (p15;q21)	1/24 (4.2)	Hama et al. (2008)

### Co-occurrence patterns of additional somatic mutations other than GATA1s

Transformation of TAM to ML-DS often arises on the background of activating signaling mutations interacting with deregulated epigenetic modifiers. For instance, there is a significant co-occurrence of variants in genes encoding tyrosine kinases and RAS proteins with variants in epigenetic regulators or cohesins both in ML-DS mouse models and patient samples (Labuhn et al., 2019). *CTCF* and *EZH2* mutations alone are insufficient to drive ML-DS in the presence of trisomy 21 and *GATA1s*, implying other somatic mutations are required (Wagenblast et al., 2021). The frequent co-occurrence of variants in *EZH2* and *CB1* is identified in a murine model of ML-DS, while *NF1* mutations appear mutually exclusive with *CB1*, *EZH2*, and *CTCF* variants (Labuhn et al., 2019). Co-occurrence of additional mutations is important for leukemic progression, but their patterns, functional effects, and clinical significance need further investigation.

## Mutational landscape of relapsed myeloid leukemia associated with Down syndrome

ML-DS usually has a low incidence of relapse, seen in approximately 5–6% of patients in developed countries, mostly because the initial disease is very sensitive to chemotherapy (Uffmann et al., 2017). However, when relapse occurs in ML-DS patients, the prognosis is less favorable. Little is known about the molecular underpinnings leading to relapse and current treatment options are less effective in relapsed patients. One study showed that in a cohort of 170 pediatric patients with ML-DS, five of 7 relapsed cases harbored trisomy 8, while the other two carried isochromosome 7 and additional material on chromosome 16 respectively (Uffmann et al., 2017). As for somatic mutations, the sequencing data from one paired sample (diagnostic and relapsed) demonstrated the presence of EZH2 F562S, JAK2 V617F and MTNR1B R316H in the relapsed sample but not at the time of diagnosis, while SMC1A R711Q, MPL W515S, JAK2 F694S and EZH2 H206fs were detected at both time-points (Labuhn et al., 2019). A lot more work will need to be done in this area in the future.

## Novel therapeutic targets

As the genomic, epigenomic and transcriptomic changes are uncovered in TAM and ML-DS, new molecular targets for prevention and treatment are being proposed. Mutations in signaling effectors are one of the most frequent events in ML-DS associated with the overactivation of pathways such as JAK-STAT, RAS/MEK/ERK and PI3K/PKB (Labuhn et al., 2019). Inhibition of these pathways may help treat ML-DS. FDA-approved JAK1/2 inhibitors, ruxolitinib and momelotinib (Sureau et al., 2021), could be considered for patients with activating JAK-STAT mutations. Similarly, drugs targeting RAS and PI3K/PKB signaling could be trialed in children with mutations in these pathways (De Castro et al., 2021). CD117/KIT expression is a marker of *GATA1s*-induced pre-leukemia- and *GATA1s/STAG2*-knock-out-induced leukemia-initiating cells. The maintenance and expansion of those cells rely on KIT signaling; thus, KIT inhibitors have emerged as potential therapeutic targets (Wagenblast et al., 2021). Further, mutations in cohesin subunits and cohesin regulators are crucial for ML-DS pathogenesis (Labuhn et al., 2019), thus targeting cohesin-mutant cells has been suggested to be a new therapeutic strategy. There are three distinct approaches through which cohesin mutated cells can be targeted: 1) direct modulation of cohesin subunits and its regulators; 2) targeting cohesin-induced deregulated signaling; and 3) targeting altered DNA damage repair mechanisms (Antony et al., 2021). STAG1 inhibition may be a suitable therapy for patients with *STAG2* mutations because it is synthetically lethal with *STAG2* variants. ML-DS displays frequent gains of DNA methylation, thus epigenetic therapies may be useful. In support, lysine-specific demethylase inhibitor T-3775440 inhibits growth of patient-derived blasts *ex vivo* (Labuhn et al., 2019). Finally, three HSA21 miRNAs (miR-99a, miR-125b and miR-155) are overexpressed in blast cells from ML-DS, and their blockage inhibits *GATA1s*-induced pre-leukemia development (Wagenblast et al., 2021). Hence, miRNAs could also become potential therapeutic targets in the future.

## Conclusions and future directions

ML-DS has three major molecular features: trisomy 21, *GATA1s* mutations, and tertiary alterations (Figure 1). Trisomy 21 drives megakaryocytic expansion through the increased gene dosage effect, but trisomy 21 may not be required for progression to ML-DS. *GATA1s* mutations are acquired during fetal liver hematopoiesis in susceptible HSCs with high proliferative potential, which leads to abnormal megakaryocytic proliferation and impaired erythroid differentiation. *GATA1s* effects begin in an immunophenotypically distinct population of fetal erythro-megakaryocytic cells. The development of ML-DS requires acquisition and selection of clones with additional somatic mutations and chromosomal structural abnormalities. Substantial progress has been made over the last 20 years in the molecular characterization of ML-DS, but some important questions remain unanswered. How do trisomy 21, *GATA1s*, additional somatic mutations and chromosomal alterations cooperate to drive ML-DS? In the context of trisomy 21 and *GATA1s*, what is the relevant co-occurrence pattern of somatic mutations and cytogenetic changes? What is the biological role and the clinical impact of such changes? Recent application of CRISPR/Cas9 technology in iPSC-based models of ML-DS started to provide some essential answers to these questions.

From the clinical standpoint, new therapies are needed for children with refractory and relapsed disease, in particular as high-dose chemotherapy causes unacceptable toxicity in DS children. To test emerging therapeutic targets, we need to advance pre-clinical disease models of ML-DS. Chromosome 21-encoded proteins and miRNAs are important players in ML-DS. Is it possible to target these molecules alone or do we need to simultaneously target secondary and tertiary genetic changes to control leukemia growth? The work needs to continue to better elucidate disease mechanisms and to develop more effective therapies.
